# Current News

**Published:** 1892-06

**Authors:** 


### Current News.

### THE WORLD'S COLUMBIAN DENTAL CONGRESS.

[The following is taken from the full report of the proceedings of the General Executive Committee, prepared by Dr. A. O. Hunt, Secretary.—Ed.]

### CONSTITUTION, CODIFIED RULES, AND COMMITTEES AS ORGANIZED AND CONFIRMED TO DATE.

NAME.

*Article I.* The World's Columbian Dental Congress.

PLACE OF MEETING.

*Article II.* Chicago, Illinois.

TIME OF MEETING.

*Article III.* Monday, August 17, 1893, continuing until August 27, 1893.

OBJECTS.

*Article IV.* The bringing together for professional, scientific, and social purposes the dentists of the United States and all other countries.

OFFICERS.

*Article V.* The officers shall be a President, four Vice-Presidents, Secretary-General, and two Assistant Secretaries (to be selected with reference to linguistic attainments), and a Treasurer.

MEETING OF COMMITTEE.

*Article VI.* The General Executive Committee shall hold its meetings for the transaction of such business at such times and places as they may elect from time to time.

DUTY OF THE COMMITTEE.

*Article VII.* It shall be the duty of the Chairman of this committee to preside at all meetings of the committee, and to carry out any matters put in charge by the General Committee. He shall be considered an *ex-officio* member of all committees, and as chief executive officer of the General Executive Committee. He shall have power, upon consulting with three members of the committee, to call a meeting, when in his judgment it may be necessary.

DUTY OF THE SECRETARY.

*Article VIII.* The duties of the Secretary of this committee shall be to keep a record of all proceedings; to receive and put in proper form, for the use of this committee, a synopsis of the work accomplished, from time to time, by each of the special committees.

DUTY OF THE TREASURER.

*Article IX.* It shall be the duty of the Treasurer to receive and have in charge all money for or belonging to the "World's Columbian Dental Congress," and to pay out the same on the order of the Chairman and the Secretary of this committee, all bills having been passed upon by the Auditing Committee.

THE POWER OF THE COMMITTEE.

*Article X.* The General Committee shall have and exercise supreme control and direction in all matters pertaining to the organization and work in 1893.

QUORUM.

*Article XI.* Eight members will be required to constitute a quorum for business.

AMENDMENTS.

*Article XII.* These rules and regulations may be amended at any regular meeting of this committee, upon a vote of two-thirds of the members of the General Committee.

*Special Committees.*—1. A General Finance Committee, to consist of three members. 2. A Programme Committee, to consist of five members. 3. A Committee on Exhibits, to consist of three members. 4. A Committee on Transportation, to consist of three members. 5. A Committee on Reception, to consist of fifteen members. 6. A Committee on Registration, to consist of seven members. 7. A Committee on Printing Transactions, to consist of three members, of which the Secretary-General shall be Chairman. 8. A Committee on Conference with State and Local Societies, with a view of eliciting their interest and co-operation. 9. A Committee on the History of Dental Legislation in this and other countries. 10. Auditing Committee, to consist of three persons, of which the Chairman of the Finance Committee shall be one. 11. A Committee on Invitation, to consist of five members. 12. A Committee on Membership, to consist of five members. 13. A Committee on Educational and Literary Exhibit, to consist of five members. 14. A Committee on Clinics in Operative Dentistry and Oral Surgery, to consist of five members. 15. A Committee on Prosthetic Dentistry, to consist of five members. 16. A Local Committee of Arrangements, to consist of five members. 17. A Committee on Essays, to consist of five members. 18. A Committee on History of Dentistry in the United States, to consist of seven members. 19. A Committee on Nomenclature, to be filled at next meeting. 20. A Committee to promote the Appointment of Dental Surgeons in the Armies and Navies of the World, to consist of fifteen members. 21. A Committee on the Care of the Teeth of the Poor, to consist of five members. 22. A Committee on Biology and Bacteriology, to consist of nine members. 23. A Committee on Prize Essays, to consist of three members. 24. An Editorial Committee, to consist of five members.

*Codified Rules.*—The admission fee to the World's Columbian Dental Congress shall be fixed at ten dollars. To be collected only from residents of the United States.

*Duties of Committee on Invitation.*—The duties of the Committee on Invitation shall be to invite such scientific persons residing in the United States and foreign countries who are not members of the profession, but who, by their recognized attainments in special departments of science, would add interest to the meeting. They shall also furnish the Committee on Membership with a list of the names and residences of those invited.

*Duties of Committee on Membership.*—The duties of the Committee on Membership shall be to pass upon all applications for membership which may be referred to them by the Committee on Registration or the Treasurer.

*Membership.*—The membership shall consist of legally-qualified and reputable dentists (as defined in the Code of Ethics of the American and Southern Dental Association) residing in the United States, and all foreign dentists regularly qualified by the laws of the countries from which they come, and such other scientific persons as may be invited by the Committee on Invitation. Each and every member to be entitled to one copy of the transactions.

The Committee on Essays shall invite suitable persons to prepare papers for presentation to the meeting.

All papers to be read before the Congress shall be in the hands of the Committee on Printing Transactions at least six months before the date of meeting, and shall not exceed forty-five minutes in time of presentation. Said committee shall have full power to accept or reject any papers, to revise or suggest a revision by the authors, and to publish or not in the transactions the whole or parts of papers read, or abridgments thereof.

NOTE.—As the General Executive Committee did not define the duties of the several committees appointed, the Secretary deems it advisable to make the following suggestions in regard to the duties of committees not already defined, subject to the action of the Executive Committee at its next meeting:

*No.* 3. *Committee on Exhibits.*—It is expected that this committee will take charge of the different exhibits of the dealers and any other exhibits of a like character.

*No.* 4. *Committee on Transportation.*—The duty of this committee will be to look after the railroad and steamship lines, and obtain the best rates possible for the members of the Congress both in this and other countries.

*No.* 5. *Committee on Reception.*—It is expected that this committee will take charge of the comfort of all members of the Congress, and especially those members and strangers from foreign countries.

*No.* 6. *Committee on Registration.*—Duties of this committee will be to register all persons applying for membership in the Congress who are entitled to the same.

*No.* 9. *Committee on the History of Dental Legislation in this and other Countries.*—It is expected that this committee will collect the data relating to dental legislation in all the countries of the world from the beginning to the present time, and present a full report to the Congress.

*No.* 10. *Auditing Committee.*—Duties of this committee will be the auditing of all accounts and bills of the Congress preparatory for the signatures of the Chairman and Secretary of the General Executive Committee.

*No.* 13. *Committee on Educational and Literary Exhibits.*—It is desired to have exhibits of a character that will show the results obtained by the various methods of dental education both in the past and up to the present time. Also an exhibit of the literary work of the profession to date.

*No.* 14. *Committee on Clinics in Operative Dentistry and Oral Surgery.*—It is expected that this committee will make every effort to secure operators to demonstrate any important or new methods of practice.

*No.* 15. *Committee on Prosthetic Dentistry.*—The duties of this committee will be of the same character as that of Committee No. 14, only the work will be confined to prosthetic dentistry.

*No.* 16. *Local Committee on Arrangements.*—Duties of this committee will be to make arrangements for the accommodation of the various committees and clinics and perform other duties of a like character, and to work in connection with the World's Congress Auxiliary.

*No.* 18. *Committee on the History of Dentistry in the United States.*—The appointment of this committee seemed necessary and important from the fact that in the work of Committee No. 8 there was collected a considerable amount of matter that should properly come under the management of a committee like No. 18. So it was deemed best to appoint a new committee to work with No. 8, and take charge of the special matter of the History of Dentistry in the United States.

*No.* 19. Not appointed.

*No.* 20. *Committee to promote the Appointment of Dental Surgeons in the Armies and Navies of the World.*—It is probably well known by the profession that for a number of years efforts have been made to appoint dental surgeons in the army of the United States,—so far without success; but it was deemed a matter of sufficient importance to appoint a committee to take charge of the matter, who might, by an organized effort, obtain at least the data that would have an influence upon any movement in this direction, should it be desirable to take action now or at some future time.

*No.* 21. *Committee on the Care of the Teeth of the Poor.*—It is common for the members of the medical profession in large cities, or in localities where it seems desirable, to devote a certain amount of their time in the interest of charity to relieve the diseases of the poor. It seems proper and right that the members of the dental profession should also do something in this way. The Executive Committee think that efforts should be made to suggest some practical plan to secure such services for the poor.

*No.* 22. *Committee on Biology and Bacteriology.*—Suggestions for the work of this committee hardly seem necessary while under the direction of its Chairman, whose experience and knowledge of results that can be obtained from work of this character are beyond question.

*No.* 23. *Committee on Prize Essays.*—It was decided by the Executive Committee of the Congress to offer a medal for the best paper on Dental Hygiene for public distribution. This committee was appointed to take charge of this matter, and its duties will be to decide upon the merits of the papers submitted, and to whom the medal shall be given.

*No.* 24. *Editorial Committee.*—The duty of this committee will be to prepare matter for public distribution and for publication in the dental journals.

The official languages of the Congress shall be English, French, Spanish, and German, and the papers shall be printed in the transactions in the language in which they are read.

After a paper has been accepted the committee shall prepare a brief synopsis to be published in the official languages of the Congress.

The Chairman of each committee shall send reports of progress to the Chairman and Secretary of the General Executive Committee at such frequent intervals as will keep them informed of all the work accomplished.

All public announcements for the General Executive Committee shall bear the signature of both the Chairman and Secretary.

All circulars issued by any committee must be sent to each member of the General Executive Committee, and they shall be of uniform size,—viz., that of the minute-forms issued by the Secretary.

*Resolved*, That this Dental Congress offer a medal for the best popular paper on Dental Hygiene, for public distribution. To be referred to Committee No. 23, to be called a Committee on Prize Essays.

The following rules for the action of the Finance Committee were adopted:

*Resolved*, That the General Finance Committee be authorized to guarantee to contributors who would be eligible to membership as follows:

1. That every contributor of twenty dollars should receive the transactions of the Congress if he does not attend; and if he attends and joins the Congress, his receipt for contributions shall be accepted by the Treasurer as full payment of the membership fee of ten dollars.

2. That every contributor of thirty dollars or upwards shall have all the advantages of the contributor of twenty dollars, and, in addition, shall receive free the commemorative medal which will be struck.

3. That every contributor of ten dollars shall receive the transactions if he does not attend; but will be expected to pay the membership fee of ten dollars in addition should he attend the Congress.

4. That any funds which may remain in the treasury after the payment of all obligations will be donated to the Dental Protective Association of the United States.

The General Finance Committee was authorized to appoint a Sub-Finance Committee for each State and Territory.

*Resolved*, That all matters of business presented at the general sessions of the Congress shall be referred to the General Executive Committee, and must receive the endorsement of this committee before they can be entertained by the President of the Congress. Carried.

*Resolved*, Whereas, the management of the World's Congress Auxiliary of the Columbian Exposition, having offered suitable accommodations in the Art Building, on the lake-front in Chicago, for the sessions of the World's Columbian Dental Congress, August 17, 1893; therefore be it

*Resolved*, That the Executive Committee gratefully accept the same in the spirit in which it is offered. And be it further

*Resolved*, That the Secretary of the committee transmit to President Bonney a copy of these resolutions as an expression of their cordial appreciation of such generosity in our behalf.

At the request of President Bonney, a committee of conference for the World's Congress Auxiliary was appointed.

*Resolved*, Whereas, all other convocations to be held in Chicago in 1893 in connection with the World's Columbian Auxiliary are called Congresses; therefore be it

*Resolved*, That the word "Meeting" be stricken out and the word "Congress" inserted in Articles 1 and 9 of the Constitution, and wherever it occurs in the proceedings to date, with the distinct understanding that this Congress shall not be considered as having connection with any preceding or future Dental Congress, but be a unique, complete, and final meeting. Unanimously adopted.

### COMMITTEES AS APPOINTED AND CONFIRMED TO DATE.

*General Executive Committee.*—Dr. W. W. Walker, Chairman, No. 67 West Ninth Street, New York City; Dr. A. O. Hunt, Secretary, Iowa City, Ia.; Dr. John S. Marshall, Treasurer, No. 9 Jackson Street, Chicago, Ill.; Dr. W. J. Barton, Paris, Texas; Dr. L. D. Carpenter, Atlanta, Ga.; Dr. J. Y. Crawford, Nashville, Tenn.; Dr. M. W. Foster, No. 9 Franklin Street, Baltimore, Md.; Dr. A. W. Harlan, No. 70 Dearborn Street, Chicago, Ill.; Dr. H. J. McKellops, No. 2630 Washington Avenue, St. Louis, Mo.; Dr. G. W. McElhaney, Columbus, Ga.; Dr. H. B. Noble, New York Avenue, Washington, D. C.; Dr. John C. Storey, Dallas, Texas; Dr. C. S. Stockton, Newark, N. J.; Dr. L. D. Shepard, No. 330 Dartmouth Street, Boston, Mass.; Dr. J. Taft, Seventh Street, Cincinnati, Ohio.

*Committee of Conference for World's Congress Auxiliary.*—W. D. Miller, F. Busch, Berlin, Germany; Thomas W. Evans, E. Magitot, E. Lecaudy, Paris, France; G. W. Sparrock, Lima, Peru; W. B. Macleod, Edinburgh, Scotland; A. W. W. Baker, Dublin, Ireland; Ernest Sjoberg, Stockholm, Sweden;

Charles S. Tomes, W. H. Coffin, London, England; W. George Beers, Montreal, Canada; H. C. Edwards, Madrid, Spain; L. D. Shepard, Boston; J. G. VanMarter, Rome, Italy; Plattschick, Pavia, Italy; Joseph Arkövy, Buda-Pesth, Hungary; C. Redard, Geneva, Switzerland; W. H. Morgan, J. Y. Crawford, Nashville, Tenn.; W. H. Dwinelle, Benjamin Lord, A. L. Northrop, W. W. Walker, New York City; R. B. Winder, F. J. S. Gorgas, M. W. Foster, Baltimore, Md.; Elisha G. Tucker, Boston, Mass.; W. W. H. Thackston, Farmville, Va.; J. B. Rich, R. Finlay Hunt, H. B. Noble, Washington, D.C.; J. D. White, J. E. Garretson,[1] Philadelphia, Pa.; W. H. Eames, H. J. McKellops, St. Louis, Mo.; J. B. Patrick, Charleston, S. C.; C. C. Knowles,[1] San Francisco, Cal.; G. V. Black, Jacksonville, Ill.; E. Bacon, Portland, Me.; W. W. Allport, A. W. Harlan, J. S. Marshall, Chicago, Ill.; W. J. Barton, Paris, Texas; J. Taft, Cincinnati, O.; C. S. Stockton, Newark, N. J.; A. O. Hunt, Iowa City, Ia.; George W. McElhaney, Columbus, Ga.; J. C. Storey, Dallas, Texas; L. D. Carpenter, Atlanta, Ga.

*No* 1. *General Finance Committee.*—L. D. Shepard, Chairman, No. 330 Dartmouth Street, Boston, Mass.; T. W. Brophy, No. 96 State Street, Chicago, Ill.; A. L. Northrop, New York City.

*No.* 2. *Programme Committee.*—Not appointed.

*No.* 3. *Committee on Exhibits.*—Charles Pruyn, Chairman, No. 70 Dearborn Street, Chicago, Ill.; Arthur E. Matteson, No. 3700 Cottage Grove Avenue, Chicago, Ill.; E. M. S. Fernandez, No. 103 State Street, Chicago, Ill.

*No.* 4. *Committee on Transportation.*—F. H. Gardiner, Chairman, No. 126 State Street, Chicago; V. H. Jackson, No. 240 Lenox Avenue, New York City; George Eubank, Birmingham, Ala.

*No.* 5. *Committee on Reception.*—W. W. Allport, Chairman, No. 9 Jackson Street, Chicago; W. W. H. Thackston, Farmville, Va.; G. H. Bently,[1] No. 70 Dearborn Street, Chicago; E. M. S. Fernandez, No. 103 State Street, Chicago; George A. Christmann, *Staats Zeitung* Building, Chicago; James McManus, No. 32 Pratt Street, Hartford, Conn.; Elisha G. Tucker, Boston, Mass.; John D. Thomas, No. 912 Walnut Street, Philadelphia; H. J. McKellops, No. 2630 Washington Avenue, St. Louis; L. L. Dunbar, No. 500 Sutter Street, San Francisco, Cal.; V. E. Turner, Raleigh, N. C.; Joseph Bauer, No. 130 Esplanade Street, New Orleans, La.; J. F. P. Hudson, No. 19 West Thirty-ninth Street, New York City; W. P. Dickinson, No. 608½ Nicollett Avenue, Minneapolis, Minn.; C. F. W. Holbrook, No. 34 Park Street, Newark, N. J.; W. J. Foster, No. 9 West Franklin Street, Baltimore, Md.; R. M. Sanger, East Orange, N. J.

*No.* 6. *Committee on Registration.*—Fred A. Levy, Chairman, No. 343 Main Street, Orange, N. J.; E. L. Clifford, No. 401 West Monroe Street, Chicago, Ill.; George N. West, No. 34 Monroe Street, Chicago, Ill.; J. Y. Crawford, Nashville, Tenn.; C. V. Rosser, Atlanta, Ga.; T. L. James, Fairfield, Ia.; W. H. Fundenberg, No. 323 Pennsylvania Avenue, Pittsburg.

*No.* 7. *Committee on Printing Transactions.*—Not appointed.

*No.* 8. *Committee on Conference with State and Local Societies.*—J. Taft, Chairman, Cincinnati, O.

LIST OF STATE COMMITTEES.

*Alabama.*—E. S. Chisholm, Chairman, Tuscaloosa; A. Eubank, Birmingham; Charles P. Robinson, Mobile; G. M. Rousseau, Montgomery.

*Arizona.*—L. N. Goodrich, Chairman, Phœnix; D. Pentland, Prescott; J. Hardy, Phœnix; W. Warnekross, Tombstone.

*Arkansas.*—M. C. Marshall, Chairman, Little Rock; W. B. Pollard, Hot Springs; L. K. Land, Pine Bluff; R. D. Seals, Fort Smith; A. E. Kimmons, Forth Smith.

*California.*—C. L. Goddard, Chairman, San Francisco; W. J. Younger, San Francisco; E. L. Townsend, Los Angeles.

*Colorado.*—P. T. Smith, Chairman, Denver; W. E. Griswold, Denver; H. P. Kelly, Denver; R. B. Weiser, Georgetown.

---

[1] Declined.

*Connecticut.*—E. S. Gaylord, Chairman, New Haven; James McManus, Hartford; R. W. Browne, New London.

*Delaware.*—C. H. Gilpin, Chairman, Middletown; C. R. Jefferis, Wilmington.

*District of Columbia.*—Henry C. Thompson, Chairman, Washington; R. B. Donaldson, J. Hall Lewis, L. C. F. Hugo, H. M. Schooley.

*Florida.*—J. N. Jones, Chairman, Jacksonville; James Chace, Ocala; Duff Post, Tampa; I. J. Welch, Pensacola.

*Georgia.*—S. B. Barfield, Chairman, Macon; Joseph H. Coyle, Thomasville; H. H. Johnson, Macon; W. C. Wardlaw, Augusta.

*Idaho.*—E. L. P. Ector, Chairman, Moscow; John H. McCallie, Moscow; A. Boston, Lewiston.

*Illinois.*—W. H. Taggart, Chairman, Freeport; C. N. Johnson, Chicago; J. J. Jennelle, Cairo.

*Indiana.*—J. B. Morrison, Chairman, Indianapolis; P. G. C. Hunt, Indianapolis; S. B. Browne, Fort Wayne.

*Iowa.*—C. J. Peterson, Chairman, Dubuque; S. C. Hatch, Sioux City; L. K. Fullerton, Waterloo.

*Kansas.*—L. C. Wasson, Chairman, Topeka; C. E. Esterley, Lawrence; William H. Schulze, Atchison.

*Kentucky.*—C. G. Edwards, Chairman, Louisville; Charles E. Dunn, Louisville; F. Peabody, Louisville.

*Louisiana.*—C. E. Kells, Jr., Chairman, New Orleans; Joseph Bauer, New Orleans; Andrew G. Friedericks, New Orleans.

*Maine.*—D. W. Fellows, Chairman, Portland; Edmund C. Bryant, Pittsfield; Henry A. Kelly, Portland.

*Maryland.*—E. P. Keech, Chairman, Baltimore; A. J. Volck, Baltimore; Edward Nelson, Frederick.

*Massachusetts.*—D. M. Clapp, Chairman, Boston; W. H. Potter, Sec'y; Eugene H. Smith, Boston; S. G. Stevens, Boston; D. B. Ingalls, Clinton; R. R. Andrews, Cambridge.

*Michigan.*—C. S. Case, Chairman, Jackson; George L. Field, Detroit; F. L. Owen, Grand Rapids.

*Minnesota.*—T. E. Weeks, Chairman, Minneapolis; M. G. Jenison, Minneapolis; C. H. Robinson, Wabasha.

*Mississippi.*—Morgan Adams, Chairman, Sardis; R. K. Luckie, Holly Springs; J. D. Miles, Vicksburg; G. B. Clements, Macon.

*Missouri.*—C. L. Hungerford, Chairman, Kansas City; A. H. Fuller, St. Louis; J. D. Patterson, Kansas City.

*Montana.*—C. S. Whitney, Miles City.

*Nebraska.*—H. T. King, Chairman, Fremont; A. W. Nason, Omaha; H. C. Miller, Grand Island; H. J. Cole, Norfolk; I. W. Funck, Beatrice.

*Nevada.*—A. Chapman, Chairman, Virginia City; M. A. Greenlaw, Reno; S. S. Southworth, Carson City.

*New Hampshire.*—C. W. Clements, Chairman, Manchester; G. A. Young, Concord; William Jarvis, Claremont; W. R. Blackstone, Manchester; C. H. Hayward, Peterborough; B. C. Russell, Keene.

*New Jersey.*—S. C. G. Watkins, Chairman, Mont Clair; B. F. Luckey, Paterson; R. M. Sanger, East Orange.

*New York.*—John I. Hart, Chairman, New York City; K. C. Gibson, New York; W. Carr, New York; M. L. Chaim, New York; Charles Butler, Buffalo; F. A. Remington, New York.

*North Carolina.*—V. E. Turner, Chairman, Raleigh; J. H. Durham, Wilmington; J. F. Griffith, Salisbury.

*North Dakota.*—S. J. Hill, Chairman, Fargo; S. P. Johnson, Grand Forks; W. O. DePuy, Bismarck; H. S. Sowles, Wahpeton; E. M. Pierce, Hillsboro.

*Ohio.*—D. R. Jennings, Chairman, Cleveland; H. F. Harvey, Cleveland; M. H. Fletcher, Cincinnati; L. E. Custer, Dayton; A. F. Emminger, Columbus.

*Oklahoma Territory.*—D. A. Peoples, Chairman, Guthrie; G. F. Dean, Oklahoma City; J. S. Nickolson, El Reno.

*Oregon.*—S. J. Barber, Chairman, Portland; E. G. Clark, Portland.

*Pennsylvania.*—L. A. Faught, Chairman, Philadelphia; C. S. Beck, Wilkesbarre; J. A. Libbey, Pittsburg.

*South Carolina.*—Thomas T. Moore, Chairman, Columbia; W. S. Brown, Charleston; A. P. Johnstone, Anderson; B. H. Teague, Aiken.

*South Dakota.*—O. M. Huestis, Chairman, Aberdeen; C. W. Stutenroth, Watertown; F. W. Blomily, Sioux Falls.

*Tennessee.*—H. W. Morgan, Chairman, Nashville; B. S. Byrnes, Memphis; W. H. Richards, Knoxville; H. E. Beach, Clarksville.

*Texas.*—W. R. Clifton, Chairman, Waco; G. M. Patten, Galveston; Tom Robinson, Houston; George S. Staples, Sherman; T. L. Westerfield, Dallas; H. J. McBride, Tyler.

*Utah.*—A. S. Chapman, Chairman, Salt Lake City; A. B. Dunford, Salt Lake; F. W. Baker, Ogden.

*Vermont.*—G. F. Cheney, Chairman, St. Johnsbury; Thomas Mound, Rutland; R. M. Chase, Bethel.

*Virginia.*—J. Hall Moore, Chairman, Richmond; W. W. H. Thackston, Farmville; Joseph R. Woodley, Norfolk; E. P. Beadles, Danville; T. H. Parramore, Hampton; D. W. Rust, Alexander.

*Washington.*—W. E. Burkhardt, Chairman, Tacoma; F. P. Hicks, Tacoma; J. C. Grasse, Seattle.

*West Virginia.*—H. H. Harrison, Chairman, Wheeling; John H. McClure, Wheeling; H. K. Jones, Parkersburg; George I. Keener, Grafton; J. N. Mahan, Charleston.

*Wisconsin.*—B. G. Marcklein, Chairman, Milwaukee; C. C. Chittenden, Madison; George H. McCausey, Janesville.

*Wyoming.*—Waiting for nominations.

*No.* 9. *Committee on the History of Dental Legislation in this and other Countries.*—William Carr, Chairman, New York City; Paul Dubois, No. 2 Rue d'Amsterdam, Paris; F. Busch, Berlin, Germany; J. H. Mummery, London, England; M. Etcheparaborda, Buenos Ayres, South America.

*No.* 10. *Auditing Committee.*—L. D. Shepard, Chairman, Boston, Mass.; R. R. Andrews, Cambridge, Mass.; Charles A. Meeker, Newark, N. J.

*No.* 11. *Committee on Invitations.*—W. C. Barrett, Chairman, No. 208 Franklin Street, Buffalo, N. Y.; E. T. Darby, No. 1513 Walnut Street, Philadelphia; S. G. Perry, No. 46 West Thirty-seventh Street, New York City; W. C. Wardlaw, Augusta, Ga.; S. W. Dennis, No 81 Flood Building, San Francisco, Cal.; Thomas H. Chandler,[1] No. 161 Newbery Street, Boston, Mass.; J. D. Patterson, Kansas City, Mo.

*No.* 12. *Committee on Membership.*—Edmund Noyes, Chairman, No. 65 Randolph Street, Chicago, Ill.; B. F. Luckey, Paterson, N. J.; E. H. Chisholm, Tuscaloosa, Ala.; C. M. Bailey, No. 28 Syndicate Block, Minneapolis, Minn.; Daniel H. McQuillen, No. 1628 Chestnut Street, Philadelphia.

*No.* 13. *Committee on Education and Literary Exhibits.*—J. J. R. Patrick, Chairman, Belleville, Ill.; J. Y. Crawford, Nashville, Tenn.; A. H. Fuller, No. 2602 Locust Street, St Louis, Mo.; C. A. Brackett, No. 102 Truro Street, Newport, R. I.; B. H. Catching, Atlanta, Ga.

*No.* 14. *Committee on Clinics in Operative Dentistry and Oral Surgery.*—C. F. W. Bödecker, Chairman, No. 60 East Fifty-eighth Street, New York City; S. C. G. Watkins, Mont Clair, N. J.; John S. Marshall, No. 9 Jackson Street, Chicago, Ill.; Arthur B. Freeman, No. 325 West Madison Street, Chicago, Ill.; H. H. Schumann, No. 240 Wabash Avenue, Chicago, Ill.; Henry W. Morgan, Nashville, Tenn.; William Crenshaw, Atlanta, Ga.

*No.* 15. *Committee on Prosthetic Dentistry.*—S. H. Guilford, Chairman, Philadelphia; L. P. Haskell, No. 211 Wabash Avenue, Chicago, Ill.; A. P. Johnstone, Anderson, S. C.; W. N. Morrison, St. Louis, Mo.; Fred C. Barlow, No. 646 Jersey Avenue, Jersey City; J. Hall Lewis, No. 1309 F Street, N.W., Washington, D. C.; A. O. Hunt, Iowa City, Ia.; R. R. Freeman, Nashville, Tenn.; E. S. Gaylord, New Haven, Conn.

---

[1] Declined.

*No.* 16. *Local Committee of Arrangements.*—E. S. Talbot, Chairman, No. 125 State Street, Chicago, Ill.; F. H. Gardiner, No. 126 State Street, Chicago, Ill.; C. N. Johnson, Opera-House Building, Chicago, Ill.; D. B. Freeman, No. 4000 Drexel Boulevard, Chicago; H. J. McKellops, No. 2630 Washington Avenue, St. Louis, Mo.

*No.* 17. *Committee on Essays.*—E. C. Kirk, Chairman, Philadelphia; J. W. Wassall, Chicago, Ill.; A. H. Thompson, Topeka, Kan.; H. H. Johnson, No. 26 Second Street, Macon, Ga.; L. G. Noel, Nashville, Tenn.

*No.* 18. *Committee on History of Dentistry in the United States.*—J. Taft, Chairman, Cincinnati, O.: Louis Jack, No. 1533 Locust Street, Philadelphia; F. T. VanWort, Brooklyn, N. Y.; F. J. S. Gorgas, Baltimore, Md.; H. L. McKellops, No. 632 Sutter Street, San Francisco, Cal.; E. G. Beatty, Cincinnati, O.; J. B. Patrick, Charleston, S. C.

*No.* 19. *On Nomenclature.*—Not appointed.

*No.* 20. *Committee to promote the Appointment of Dental Surgeons in the Armies and Navies of the World.*—M. W. Foster, Chairman, Baltimore; B. Holly Smith, Baltimore; George Cunningham, Cambridge, Eng.; De Gallippe, Paris; Adolph Weil, Munich; J. B. Wilmott,[1] Toronto, Can.; John E. Gievers, Amsterdam, Holland; E. De Trey, Vevey, Switzerland; A. Szigmondy, Vienna; O. Mela, Geneva, Italy; V. Haderup, Copenhagen; O. J. Crustchow, St. Petersburg, Russia; Alex. McG. Denham, Monjitas 68½, Chili; George B. Newland, 107 Calle Florida, Buenos Ayres.

*No.* 21. *Committee on Care of the Teeth of the Poor.*—W. J. Barton, Chairman, Pavis, Texas; C. A. Brackett, Newport, R. I.; G. S. Dean,[1] San Francisco; T. D. Ingersoll, Erie, Pa.; W. M. Fisher, Dundee, Scotland.

*No.* 22. *Committee on Biology and Bacteriology.*—R. R. Andrews, Chairman, Cambridge, Mass.; M. H. Fletcher, Cincinnati, O.; W. X. Sudduth, Minneapolis, Minn.; W. D. Miller, Berlin, Germany; J. H. Mummery, London, England; D. E. Caush, Brighton, England; E. Magitot, Paris, France; M. Morgensten, Baden Baden, Germany; George S. Allen, New York City.

*No.* 23. *Committee on Prize Essays.*—Theo. Stanley, Chairman, Kansas City, Mo.; J. Hall Moore,[1] Richmond, Va.; C. S. Stockton, Newark, N. J.

*No.* 14. *Editorial Committee.*—W. W. Walker, Chairman, New York City; A. O. Hunt, Iowa City; L. D. Shepard, Boston; J. Taft, Cincinnati; J. S. Marshall, Chicago.

*No.* 25. *Nominating Committee.*—W. W. Walker, Chairman, New York City; A. W. Harlan, Chicago, Ill.; John S. Marshall, Chicago, Ill.

---

### PENNSYLVANIA AND NEW JERSEY STATE DENTAL SOCIETIES' JOINT UNION MEETING.

TO BE HELD AT CRESSON SPRINGS, PA., JULY 19, 20, 21, 1892.

THE attention of the profession is called to the formation for this meeting of a Clinical Conference, composed of well-known practitioners, to be divided into three sections, for the presentation by dentists in actual practice for advice. Section 1. Oral Surgery,—viz., treatment of pyorrhœa alveolaris, congenital deformities, oral irregularities, etc. Section 2. Mechanical dentistry,—crown- and bridge-work, gold and porcelain crowns, metal and plastic bases,

---

[1] Declined.

etc. Section 3. Operative dentistry,—all cases in general practice. Dentists are requested to communicate at an early date with the undersigned chairmen of the State Committees.

Dr. W. E. Magill, Erie, Pa.
Dr. H. A. Hull, New Brunswick, N. J.

---

### AMERICAN DENTAL SOCIETY OF EUROPE.

The American Dental Society of Europe will hold its eighteenth meeting at Basel, Switzerland, August 1, 2, and 3. Members of the profession are cordially invited to attend. Clinics will be a special feature of this meeting. The University will place desirable rooms at the disposal of the Society, and an ingenious amphitheatre for accommodating in the immediate vicinity of the patient a larger number of spectators than are able to witness operations under ordinary circumstances will be loaned by the Swiss Dental Association. Programmes may be had on application to the President, Dr. Bryan, of Basel, or to

Chas. W. Jenkins,
*Secretary.*

---

### THE MISSOURI STATE DENTAL ASSOCIATION.

The Twenty-eighth Annual Meeting of this Association will be held at Clinton, Missouri, commencing Tuesday, July 5, and continuing four days. Members of the profession are cordially invited to be present.

William Conrad,
*Corresponding Secretary.*

St. Louis, Missouri.

---

### MASSACHUSETTS DENTAL SOCIETY.

The Twenty-seventh Annual Meeting of the Massachusetts Dental Society will be held in Boston, July 7 and 8, 1892. It is intended to make this meeting of unusual interest. The annual address is to be delivered by Dr. L. D. Shepard, of Boston. Other prominent men are expected to participate in the meeting. All members of the profession are invited to attend.

Edgar O. Kinsman,

*Secretary.*

Cambridge, Mass.

